# Presence of aberrant epididymal tubules revealing undifferentiated epithelial cells and absence of spermatozoa in a combined neuraminidase-3 and -4 deficient adult mouse model

**DOI:** 10.1371/journal.pone.0206173

**Published:** 2018-10-25

**Authors:** Regiana Oliveira, Louis Hermo, Alexey V. Pshezhetsky, Carlos R. Morales

**Affiliations:** 1 Department of Anatomy and Cell Biology, McGill University–Montreal, Canada; 2 Division of Medical Genetics, Centre Hospitalière Universitaire Sainte-Justine, University of Montréal—Montreal, Canada; Universite Clermont Auvergne, FRANCE

## Abstract

Mammalian neuraminidases are responsible for the removal of sialic acids from glycoproteins and glycolipids and function in a variety of biological phenomena such as lysosomal catabolism and control of cell differentiation and growth. Disruption of *Neu3* and *Neu4* genes has led to the generation of a mouse model revealing severe neurological disorders. In this study a morphological analysis was performed on the epididymis of 3 month-old *neu3*^*-/-*^*neu4*^*-/-*^ mice as compared with wild type animals. In *neu3*^*-/-*^*neu4*^*-/-*^ mice the majority of tubules of the main epididymal duct were large and lined by differentiated epithelial cells, but revealing lysosomal abnormalities in principal and basally located cells. Of particular note was the presence of aberrant epididymal tubules (ATs) juxtaposed next to the main tubules. ATs were small and of different shapes. Layers of myoid cells encased ATs, which they shared with those of the main tubules, but no interstitial space existed between the two. While some ATs were a dense mass of cells, others revealed a distinct lumen devoid of spermatozoa. The latter revealed an undifferentiated epithelium consisting of cuboidal cells and basal cells, with junctional complexes evident at the luminal front. The absence of spermatozoa from the lumen of the ATs suggests that they were not in contact with the main duct, as also implied by the undifferentiated appearance of the epithelium suggesting lack of lumicrine factors. Despite the presence of ATs, the main duct contained ample spermatozoa, as the *neu3*^*-/-*^*neu4*^*-/-*^ mice were fertile. Taken together the data suggest that absence of Neu3 and Neu4 leads to defects in cell adhesion and differentiation of epithelial cells resulting in aberrant tubular offshoots that fail to remain connected with the main duct. Hence Neu3 and Neu 4 play an essential role in the guidance of epithelial cells during early embryonic formation.

## Introduction

A transit time through the lumen of the efferent ducts and epididymis is crucial for transforming spermatozoa from an infertile and immotile state into cells with full fertilizing capability [1–4]. The composition of the epididymal luminal fluid bathing spermatozoa is considered one of the more complex systems in the body in terms of chemical components and physical interactions with proteins and lipids [3, 5–7].

The epithelial cells lining the epididymal duct, traditionally identified as principal, narrow, apical, clear, and basal cells, modify the composition of the epididymal lumen by their secretory and endocytic functions in addition to a protective role [6, 8–13]. In addition a population of mononuclear phagocytes (Cdc11+ dendritic cells and F4/80 macrophages) reside at the base of the epithelium along with migrating halo cells [14–17]. In each of the four major regions, i.e. initial segment, caput, corpus and cauda, these cells define the structural integrity and composition of the lumen by their unique functional signature [2, 6, 18–22]. Secretion is a major function of principal cells and involves the release of proteins that interact with the surface of spermatozoa. On the other hand, endocytosis results in the removal of proteins from the lumen, some shed by spermatozoa, and is a major function of nonciliated cells of the efferent ducts as well as epithelial epididymal clear cells [2, 23–25].

The endocytic organelles whereby proteins and other substances are removed from the lumen of the efferent ducts and epididymis have been well documented [2, 24, 26–28]. After binding to the receptor in coated pits, each protein is destined to appear in a temporal and sequential manner in early and late endosomes (multivesicular bodies) and finally lysosomes where they are degraded, a process also defined in other cell types [29–33].

In addition to proteins, other substances endocytosed by cells include plasma membrane gangliosides (sialylated glycolipids, members of a large glycosphingolipid family, consisting of sialylated glycans attached to ceramide lipids). As integral components of eukaryotic cell membranes, gangliosides play crucial cellular roles by acting as receptors for several bioactive factors and by their direct involvement in cell adhesion, migration and modulation of several cell functions including membrane trafficking, apoptosis and cell proliferation [34, 35]. The catabolism of gangliosides is an essential process for cellular homeostasis and takes place in lysosomes involving the action of several hydrolases acting in a highly orderly sequence [36, 37].

Ineffective degradation of internalization of gangliosides in lysosomes leads to a variety of lysosomal storage diseases such as seen with disruption of β-Hexosaminidase A (Hex) in the case of Tay-Sachs and Sandhoff diseases [38]. Inactivation of Hex in mice results in a dramatic alteration in the number, size and appearance of lysosomes in epithelial cells of the efferent ducts and epididymis; some take on a highly vacuolated appearance [39–42]. The structural phenotype of the epithelial epididymal cells as displayed by lysosomal accumulation is typical of other lysosomal storage diseases seen in other tissues [43–46]. Indeed similar observations have also been reported in mouse knockout models of prosaposin, also known as sulfated glycoprotein-1 (SGP-1), where in the testis, epididymis and prostate, the epithelial cells revealed a storage dysfunction in lysosomes [47].

Sialic acids are terminal acidic monosaccharides found on glycoproteins and glycolipids. Sialic acids function as crucial recognition markers in multicellular organisms where they mediate a variety of biological phenomena, including cell differentiation, interaction, migration, and adhesion. The removal of sialic acid residues from glycoconjugates in vertebrates is mediated by a family of neuraminidases (sialidases) [48, 49]. To date, four neuraminidases have been described in mammals, designated as Neu1, Neu2, Neu3 and Neu4, with each presenting distinct localizations, enzymatic properties and substrate specificities [50–52]. Neu3 modulates plasma-surface biological events and plays a pivotal role in controlling transmembrane signaling for different cellular processes, including cell adhesion, recognition and differentiation. Neu3 is also localizes to endosomes and lysosomes [53], while Neu4 is present in the lysosomal and mitochondrial lumen and is involved in lysosomal catabolism [51, 54–56].

In the central nervous system, gangliosides (sialylated glycolipids) play an essential role by regulating recognition and signaling in neurons. It has been demonstrated that Neu3 and Neu4 are active in ganglioside degradation playing important roles in catabolic processing of brain gangliosides by cleaving terminal sialic acid residues in their glycan chains [52]. In the absence of either one of these enzymes or a combined double knockout, the specific ganglioside substrate accumulates in lysosomes, leading to the development of a lysosomal storage disorder, causing severe consequences to the organism [50, 51, 54, 57–59]. The development of these conditions thus highlights the importance of Neu3 and Neu4 in the catabolism of gangliosides.

Despite the importance of neuraminidases to the cellular homeostasis of many tissues of the body, little is known about the functional significance of neuraminidases in the male reproductive tract. The presence of sialic acid-containing glycoconjugates has been described in ciliated cells of the efferent ducts and narrow, clear and basal cells of the epididymis [60]. In the case of spermatozoa, a sialic acid rich glycocalyx coats its surface, along with two neuraminidases (Neu1 and Neu3). Inhibition of neuraminidase activity interferes with the binding of spermatozoa to the zona pellucida of the oocyte indicating new insights into the dynamic remodeling of the glycocalyx of spermatozoa before fertilization [61].

However, to date little data are available on the functional significance of neuraminidases in epithelial cells of the efferent ducts and epididymis. The objective of this study was to profit from the availability of a mouse model deficient in both *Neu3* and *Neu4* genes and examine effects on epithelial cells lining the efferent ducts and epididymis of adult mice. The results obtained in this investigation revealed that inactivation of both *Neu3* and *Neu4* genes leads to an accumulation of gangliosides that affects lysosomes of the epithelial cells of the epididymis. Additionally, the presence of small aberrant tubules alongside tubules of the main epididymal duct suggest a role for Neu3 and Neu4 for proper recognition and cell adhesion of its lining cells during early embryonic formation of the epididymal duct.

## Materials and methods

### Animal preparation

Double knockout *neu3*^*-/-*^*neu4*^*-/-*^ mice were obtained by crossing *Neu4* and *Neu3* KO strains as previously described [59]. Before to experimentation, wild type (WT) and *neu3*^*-/-*^*neu4*^*-/-*^ animals were housed under controlled laboratory environment: constant temperature, light/dark cycle (12 h of light and 12 h of darkness), humidity and access to food and water *ad libitum*. All mice were bred and maintained in the Canadian Council on Animal Care (CCAC)-accredited animal facilities of the Ste-Justine Hospital Research Center according to the CCAC guidelines. Approval for the animal care and the use in the experiments was granted by the Animal Care and Use Committee of the Ste-Justine Hospital Research Center.

### Tissue preparation for light microscope (LM) and electron microscope (EM) imaging

Three month-old wild type (WT) and *neu3*^*-/-*^*neu4*^*-/-*^ mice (n = 3 for each group) were anesthetized with an intraperitoneal injection of sodium pentobarbital. Before cardiac perfusion, the efferent ducts and epididymis of the left side of each mouse were excised and immersed in Bouin’s fixative for LM immunocytochemical analyses, while these tissues on the right side of each mouse were processed for EM analysis. For the latter method, fixation was performed via the left ventricle with 2.5% glutaraldehyde in 0.1M sodium cacodylate buffer (pH 7.4) containing 0.05% calcium chloride. After 10 min of fixation, the samples were placed in fresh fixative for an additional 6 hr and then washed overnight at 4°C in 0.1M sodium cacodylate buffer (pH 7.4). On the following day, the samples were washed three times for 10 min each in cacodylate buffer, and then immersed for 2 hours in a solution of 1% osmium tetroxide and 1.5% potassium ferrocyanide. The tissues were dehydrated in a graded series of acetone and embedded in Epon. Semithin sections (0.5μm) of the tissue blocks were cut with glass knives and stained with Toluidine blue for LM analysis. Thin sections (120 nm) of selected areas of each block were cut with a diamond knife and mounted on 200-mesh copper grids. The grids were stained for 5 min with uranyl acetate and 3 min with lead citrate. Thin sections were examined at 120 kV in a FEI Tecnai 12 TEM (FEI, Hillsboro, OR, USA) located at the Facility for Electron Microscopy Research (FEMR) at McGill University. Images were collected with a AMT XR80C CCD camera (Advanced Microscopy Techniques Corp, Woburn, MA, USA). A detailed EM analysis was undertaken of the different cell types of each epididymal region and in the case of each animal.

### Immunolocalization of prosaposin

Prosaposin LM immunocytochemical localizations were performed on Bouin-fixed tissues embedded in paraffin and sectioned at 5 μm. For this purpose, sections of efferent ducts and epididymis from both WT and *neu3*^*-/-*^*neu4*^*-/-*^ mice were deparaffinized in Citrisolv (Fisher DC1601; Fisher Scientific, Ottawa, ON, Canada) and rehydrated in a series of graded ethanol solutions. After rehydration, the sections were immersed in 300 mM glycine (Sigma St Louis, MO) for 10 minutes to block free aldehyde groups. For blocking endogenous peroxidase activity, the sections were immersed in 0.03% hydrogen peroxide for 20 minutes. After washing in 0.1% Tween 20 in Tris buffer (TBST), the sections were treated with 5% bovine serum albumin (BSA) in phosphate buffered saline (PBS) for 30 minutes to block any non-specific antibody binding. The sections were then incubated for 1.5 hrs at room temperature with the primary rabbit anti-prosaposin antibody characterized and purified as described by Morales et al. [47] diluted at 1:300. For negative controls, the sections were treated with BSA 5% in PBS rather than the primary antibody. All sections were washed with TBST and incubated for 1 hr with a peroxidase conjugate secondary antibody goat anti-rabbit IgG, diluted at 1:500 (Sigma St Louis, MO, USA). The sections were washed with TBST and incubated with 3,3-diaminobenzidine tetrahydrochloride (2% v/v) (Dako, Burlington, Canada). Methylene blue was used to counterstain the sections. The sections were dehydrated in a series of graded ethanol solutions and Citrisolv and mounted with a coverslip using Permount (Fisher, SP15-100 Ottawa, ON, Canada). Immunostaining was performed in triplicate on sections of the efferent ducts and epididymis from each of the 3 animals of both groups.

## Results

### Alterations in the efferent ducts of *neu3*^*-/-*^ *neu4*^*-/-*^ mice

**Fig 1:**
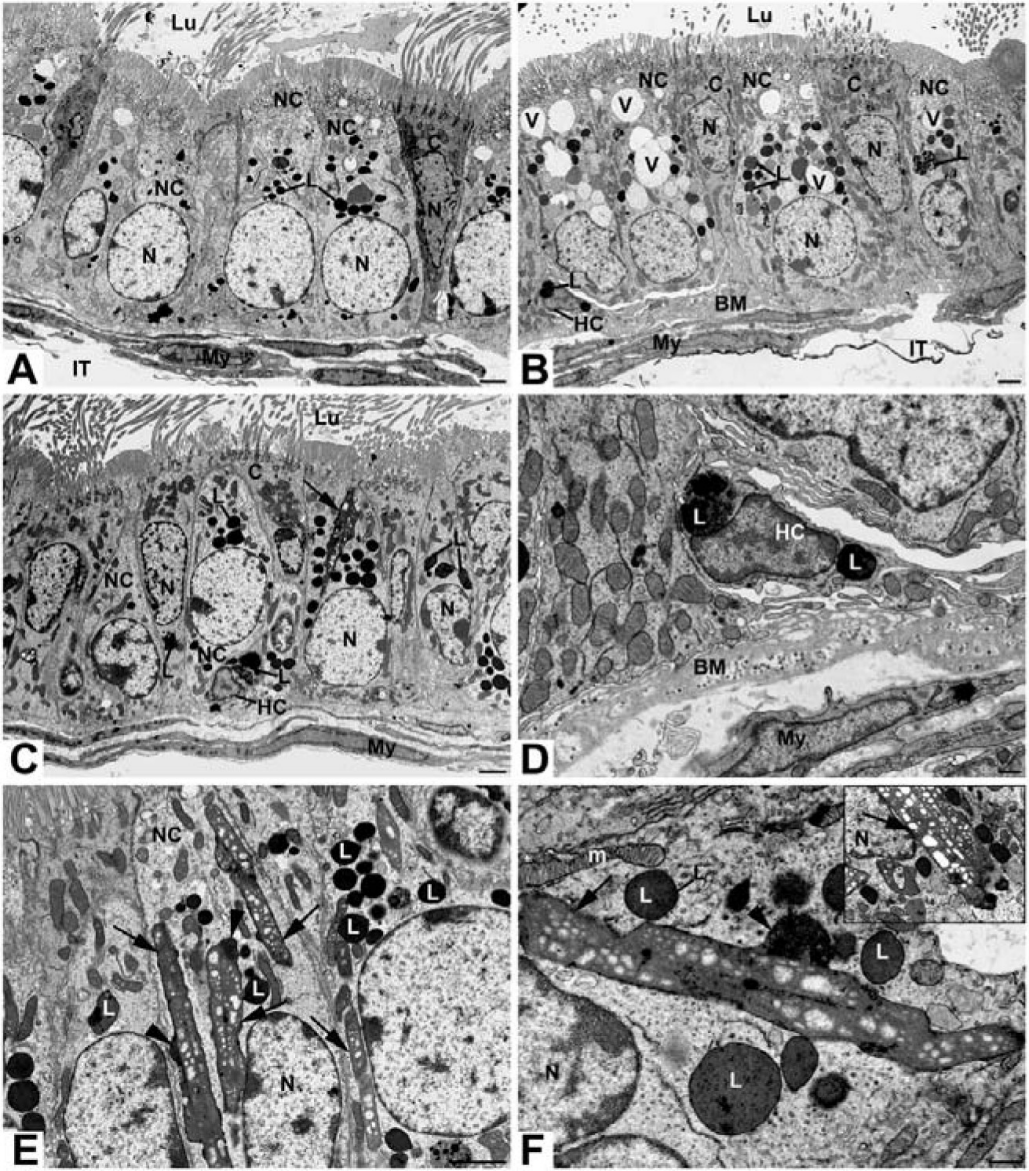
EM micrographs of the efferent ducts of WT (**A**) and *neu3*^*-/-*^*neu4*^*-/-*^ (B-F) mouse model. In (**A**), several small to medium sized dense lysosomes (L) appear in a nonciliated cell (NC), while in (**B**), many large vacuoles (V) are noted. Large, long cylindrical structures (arrows) with an irregular outline are evident in *neu3*^*-/-*^*neu4*^*-/-*^ mice, with a matrix revealing small to medium sized spaces often aligned in parallel rows (**C, E, F, inset**). Electron dense bulges (arrowheads) along the length of these cylindrical structures are noted, with an internal appearance of the dense L nearby, suggesting their fusion with the tubular structures (**E, F**). Halo cell (HC) exhibits large L (**B-D**). The basement membrane (BM) of *neu3*^*-/-*^*neu4*^*-/-*^ mice is highly convoluted and contains small vesicular profiles (**B, D**). Ciliated cells (C) are indicated. Scale bars: **A, B, C, E**: 2μm; **D, F**: 500nm.

In wild type mice (WT), the nonciliated cells of the efferent ducts displayed a few small pale stained apical endosomes and several small to medium size dense supranuclear lysosomes (Fig 1A). In *neu3*^*-/-*^*neu4*^*-/-*^ mice, numerous large pale stained bodies were additionally noted (Fig 1B). The large pale bodies occupied both the apical and supranuclear regions of the cell and were present in efferent ducts of the proximal regions situated close to the rete testis (Fig 1B). In efferent ducts of the distal regions next to the initial segment, large dense lysosomes were noted in nonciliated cells (Fig 1C). In addition, *neu3*^*-/-*^*neu4*^*-/-*^ mice revealed halo cells that were characterized by presence of large dense lysosomes (Fig 1B–1D). When present, halo cells revealed a small cytoplasm to nuclear ratio and had long processes extending for a considerable distance. They did not contact the basement membrane.

A striking feature of nonciliated cells of *neu3*^*-/-*^*neu4*^*-/-*^ mice was the presence of long large irregularly shaped membrane bound structures that had a cylindrical-like appearance (Fig 1C, 1E and 1F). Some stretched from the apical to the supranuclear region of the cell (Fig 1C), while others were seen alongside the nucleus (Fig 1E). Such cylindrical structures consisted of a moderately dense stained matrix in which pale stained areas were noticeable (Fig 1E and 1F). The latter were of variable size and often arranged in rows (Fig 1F and inset). Along the length of these cylindrical structures, small focal densely stained bodies were applied to their surface (Fig 1E and 1F). The texture of these dense bodies was not unlike the dense lysosomal elements nearby, and the cylindrical structures also contained electron dense deposits not unlike that noted in adjacent lysosomes (Fig 1F). It is conceivable that these masses corresponded to lysosomes that had fused with the cylindrical structures. It was also noted that their matrix was comparable in texture and density to that of mitochondria of the cell and that of their pale stained areas to the spaces delineated by their cristae (Fig 1E and 1F). Ciliated cells were noted in *neu3*^*-/-*^*neu4*^*-/-*^ mice, with some demonstrating enlarged lysosomes (not shown).

Another conspicuous feature of *neu3*^*-/-*^*neu4*^*-/-*^ mice was the basement membrane underlying the epithelium. While fairly smooth, uniform and thin in WT mice (Fig 1A), that of double knockout mice was highly convoluted, anastomotic, thickened and contained numerous small electron dense vesicular profiles (Fig 1B–1D). Myoid cells did not appear to be affected in *neu3*^*-/-*^*neu4*^*-/-*^ mice (not shown).

### Alterations in the epididymis of *neu3*^*-/-*^*neu4*^*-/-*^ animals

#### Light microscopy

**Fig 2:**
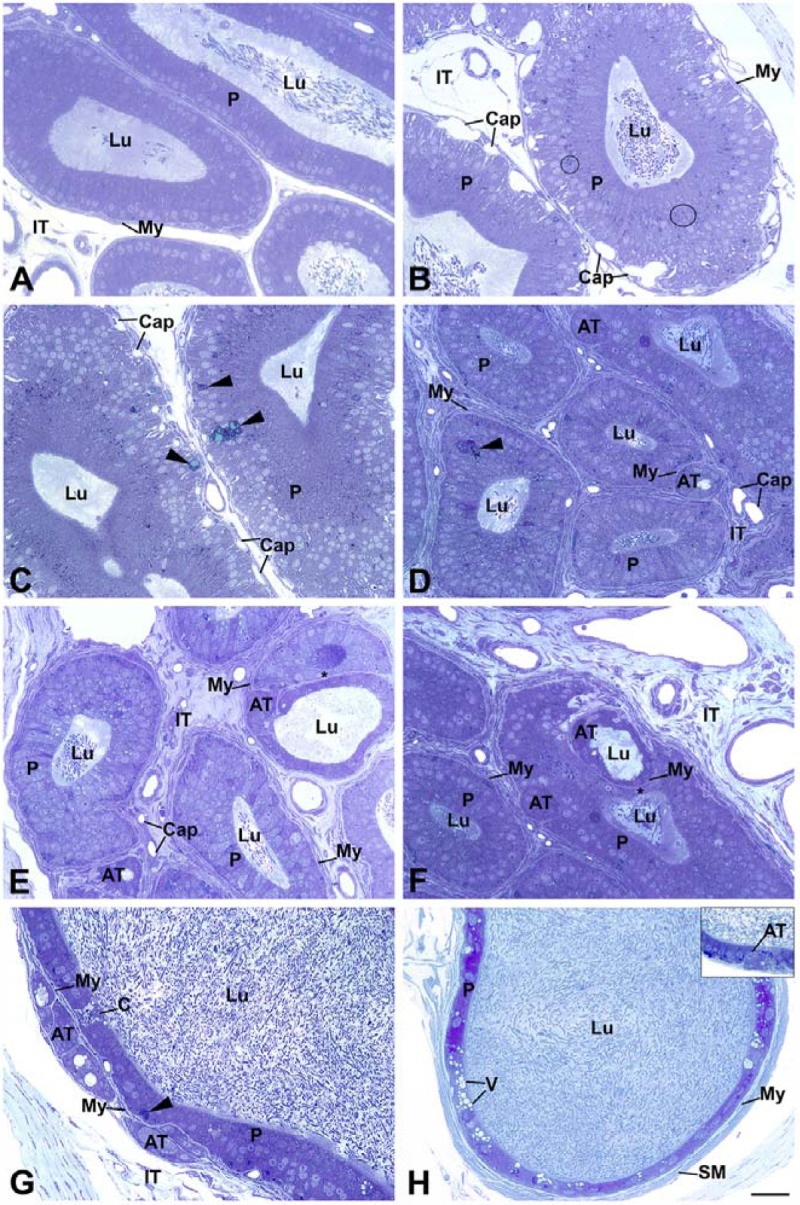
LM micrographs of semithin sections (0.5 μm) of the initial segment of WT (**A**) and initial segment (**B-C**), caput (**D-F**), and proximal (**G**) and distal (**H**) cauda regions of *neu3*^*-/-*^*neu4*^*-/-*^ mice. In (**A**), principal cells (P) are tall columnar and the lumen (Lu) is small containing spermatozoa (Spz). In (**B, C**), P cells appear to be larger and contain many small dense lysosomes (L). Capillaries (Cap) of large size are abundant between the epithelium and myoid cell (My) layers. In (**D-G**), ATs of different shapes and sizes reside in close proximity to normal tubules. Some aberrant tubules (ATs) are a dense mass of cells without a central Lu (**D, F, G**), while others reveal a Lu (**D-G**) with absence of Spz (**E, F**). ATs form deep depressions in the normal tubules (asterisks) (**E, F**). ATs with a lumen consist of undifferentiated cells with a cuboidal appearance (**E, F**). The wall of some ATs is kinked which disrupts the homogeneity of their tubular appearance (**E, F**). ATs are enveloped by My cells, which are shared with the normal tubules (**D-F**). Large dense masses (arrowheads) appear in the basal region of the epithelium (**D, G**). In the cauda region, some ATs run parallel to the main tubules (**G**), but some impinge upon it (**H inset**). P cells of the distal cauda show large vacuolar L in the cytoplasm (**H**). The Lu of normal tubules of *neu3*^*-/-*^*neu4*^*-/-*^ mice is filled with Spz. Scale bar: 20μm.

Alterations to the epithelium of *neu3*^*-/-*^*neu4*^*-/-*^ mice were noted in all regions of the epididymis as compared to WT mice. In the initial segment (IS) of WT mice (Fig 2A), principal cells were tall columnar with prominent microvilli, enveloping a small lumen containing spermatozoa. In the IS of *neu3*^*-/-*^*neu4*^*-/-*^ mice (Fig 2B and 2C), principal cells appeared to be larger than in the controls and contained numerous small dense supranuclear lysosomes and occasional large dense masses (Fig 2B and 2C). Several small and large size capillaries were abundant between the epithelium and myoid cell layer (Fig 2B and 2C). Spermatozoa were plentiful in the lumen (Fig 2B and 2C). The presence of densely stained cells of small and large size was a conspicuous feature of the double KO mice; such cells often resided at the basal area of the epithelium (Fig 2C and 2D).

A conspicuous feature of the epithelium of *neu3*^*-/-*^*neu4*^*-/-*^ mice of the caput (Fig 2D–2F) and cauda (Fig 2G and 2H) regions was the presence of aberrant tubules of different shapes and sizes that resided in close proximity to that of normal epididymal tubules, but which were significantly smaller in size. Such tubules were never encountered in WT mice (Fig 2A). Some aberrant tubules appeared as a small dense mass of cells, while others often formed a distinct tubular structure with a lumen. While spermatozoa were evident in the lumen of normal epididymal tubules, they were absent from the aberrant tubules (Fig 2D–2F). At times the aberrant tubules formed deep depressions in the normal epididymal tubules causing the epithelium at that site to be malformed and disfigured; the epithelial cells at that site were often squamous in appearance (Fig 2E and 2F). More than one aberrant tubule could be seen impinging on a given normal epididymal tubule (Fig 2F). The wall of some aberrant tubules was not uniform revealing kinks appearing to disrupt its integrity (Fig 2F). Myoid cells were enveloped by aberrant tubules and when apposed to normal tubules they were shared between the two (Fig 2D–2F). In the cauda region several aberrant tubules often lined up parallel to the main epididymal duct, with all being smaller in size than the normal tubules, but larger than those of the caput. When a lumen was apparent it was always devoid of spermatozoa (Fig 2G). Aberrant tubules of the cauda also impinged on the main duct (Fig 2G inset). In the cauda region of *neu3*^*-/-*^*neu4*^*-/-*^ mice, principal cells often contained large vacuoles in their cytoplasm as well as large dense bodies (Fig 2G and 2H).

### Structural features of epithelial cells of the initial segment, caput, corpus and cauda regions as seen with the electron microscope

#### Initial segment of the epididymis

**Fig 3:**
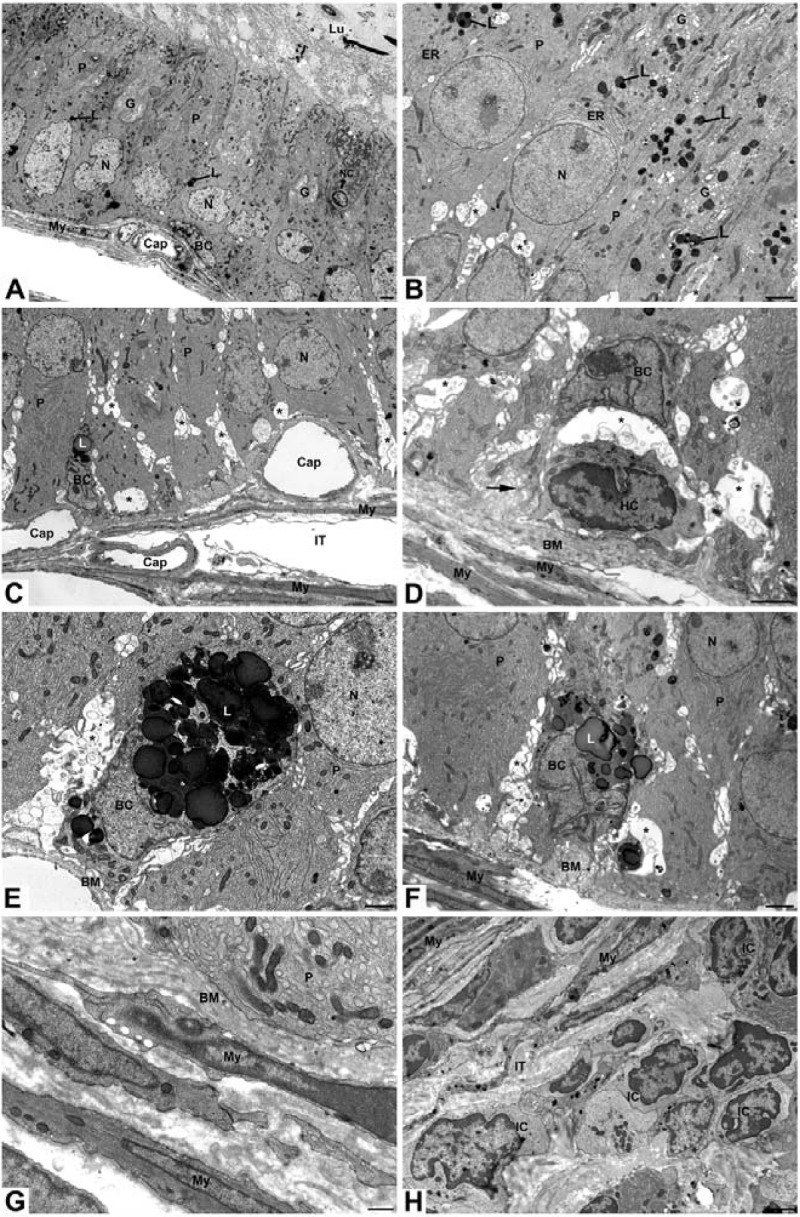
EM of the initial segment of epididymis of WT (**A**) and *neu3*^*-/-*^*neu4*^*-/-*^ mice (**B-H**) mice. In (**A**), principal cells (P) cells demonstrate an elaborate Golgi (G), small dense L and large spherical nuclei (N). A narrow cell (NC) and basal cell (BC) are indicated. A thin BM underlies the epithelium, beneath which lie several layers of My cells. A Cap is sandwiched between the BM and inner My cell layer. In (**B**), many small dense L appear in P cells as well as parallel layers of cisternae of endoplasmic reticulum (ER) next to the G. A BC contains a large lysosomal/lipidic structure (L). Large Cap reside between the BM and underlying My cells. In (**D**), basal area of epithelium shows a HC near a BC maintaining its contact with the BM by thin processes (arrow). In (**E, F**) a BC contains a gigantic L. In (**B-F**), large dilated intercellular spaces (asterisks) appear between adjacent P cells and contain numerous membranous vesicular profiles and principal cell interdigitations. In (**G**), the BM is thickened and multilayered and takes on a convoluted and anastomotic appearance. My cells appear normal. In (**H**) many small immune cells (IC) appear in the intertubular space (IT). Scale bars: **A-F, H** = 2μm; **G** = 500nm.

In the initial segment of WT mice, tall columnar principal cells demonstrated an elaborate supranuclear Golgi apparatus, several MVBs, small dense lysosomes and a more or less spherical nucleus with indentations. Narrow and basally located cells were also evident (Fig 3A). A thin basement membrane underlined the epithelium, beneath which several layers of myoid cells were noted. Capillaries were sandwiched between the basement membrane and inner myoid cell layer (Fig 3A). In *neu3*^*-/-*^*neu4*^*-/-*^mice, principal cells revealed numerous small dense lysosomes appearing in the supranuclear area. An elaborate supranuclear Golgi was evident, as well as overlying parallel layers of cisternae of endoplasmic reticulum (Fig 3B). Dilated intercellular spaces were a prominent feature of the double KO mice and resided towards the basal area of the epithelium (Fig 3B–3F). Such spaces appeared between adjacent principal cells and contained numerous membranous vesicular profiles, whorls and principal cell interdigitations (Fig 3B–3F). Basally located cells showed a variety of phenotypes. Some basally located cells contained large lysosomal/lipidic structures that at times filled their entire cytoplasm (Fig 3C, 3E and 3F), while others were small and ill defined (Fig 3D). Even when displaced from the base of the epithelium, basally located cells retained their contact with the basement membrane by thin processes (Fig 3D–3F). Halo cells were evident and often seen next to basally located cells (Fig 3D). Large dilated spaces hovered over the basally located cells and were filled with vesicular and membranous profiles (Fig 3D–3F). The underlying interstitium was greatly modified. The basement membrane was thickened and highly convoluted and multilayered (Fig 3D–3G). Capillaries larger than noted in WT mice resided between the basement membrane and underlying myoid cells; they also appeared to be more numerous (Fig 3C). Immune cells at times filled the interstitial space and this was also noted in other epididymal regions (Fig 3H).

#### Caput epididymidis

**Fig 4:**
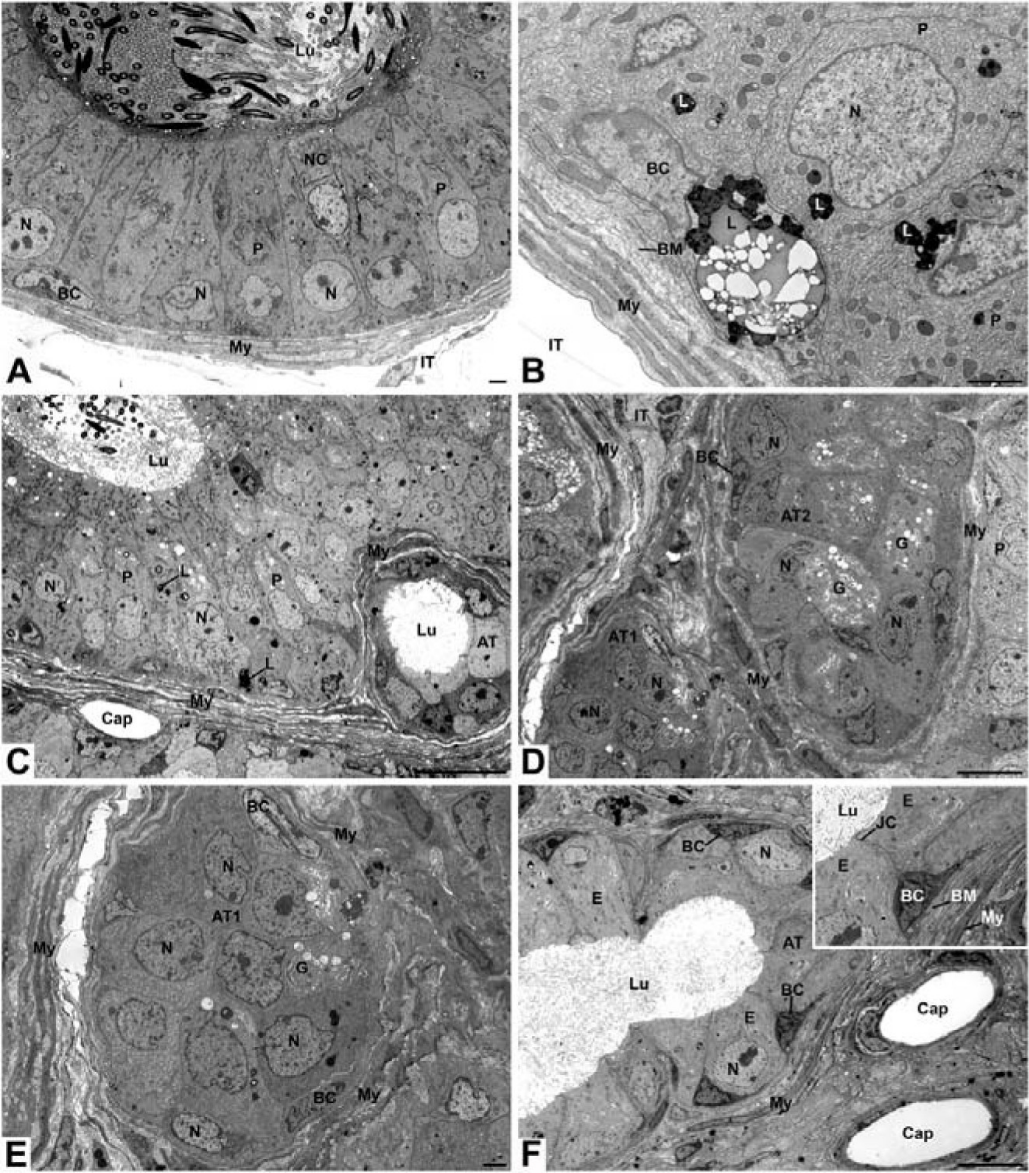
EM of the caput epididymidis of WT (**A**) and *neu3*^*-/-*^*neu4*^*-/-*^ mice (**B-F**) mice. In (**A**), P cells, BC and NC are indicated. Spz are evident in Lu. In (**B**), a BC contains a large lysosomal/lipidic structure (L) and the basal area of a P cell shows a large irregularly shaped L. In (**C**), a small AT, formed of low cuboidal undifferentiated epithelial cells, is lodged within a large indentation of a normal tubule. The AT envelops a central Lu devoid of Spz. My cells completely enclose the AT which are shared by the normal duct. A large irregularly shaped L is seen in the base of a P cell. In (**D**) two adjacent ATs are evident with one impinging on a normal tubule. In (**E**) higher mag of the tubule seen in Fig (**D**), the AT is smaller than the normal tubule, is made up of undifferentiated epithelial cells and does not shows a central lumen. Images (**F and the inset**) show an AT made up of epithelial cells (E) that extend from the BM to the central lumen (Lu). The inset is a high power of (**F**) revealing that the epithelial cells form junctional complexes (JC) at the apical extremities near the lumen. These cells show a G, but are in no way are they comparable to the highly differentiated P cells of the normal tubule. The Lu is devoid of Spz but contains sectional profiles of the epithelial cell microvilli (Mv). BC border the periphery of the ATs (**E and F)**. Scale bars: **A, B, E, F** = 2 μm; **C, D** = 10 μm.

EM of WT mice of the caput epididymidis revealed columnar principal cells with few dense small lysosomes, and narrow and basally located cells (Fig 4A). In *neu3*^*-/-*^*neu4*^*-/-*^ mice, principal cells showed a few larger lysosomal bodies scattered in the cytoplasm (Fig 4B and 4C). Basally located cells were readily apparent as they showed large dense lysosomal/lipidic structures (Fig 4B). A conspicuous feature of the caput, corpus and cauda regions was the presence of small tubules that were situated close to those of the main epididymal duct. Such tubules to be referred to as aberrant tubules revealed different morphologies. While some formed a distinct tubule with a central lumen (Fig 4C and 4F), others appeared to be a congealed mass of cells (Fig 4D and 4E). The cells forming the latter revealed some differentiated features such as a conspicuous Golgi apparatus and ER cisternae, however no lumen was evident in these tubules (Fig 4D and 4E). Aberrant tubules with a central lumen consisted of epithelial cells, which were short and cuboidal, and in no way were they comparable to that of the fully differentiated epithelial cells of tubules of the main duct (Fig 4F). The epithelial cells of aberrant tubules with a lumen were cuboidal and appeared undifferentiated without prominent organelles. However, they appeared to form junctional contact points at their apical extremities and possessed microvilli extending into the lumen (Fig 4F). It was not uncommon to find small ill defined basally located cells in the epithelium of the aberrant tubules (Fig 4F). WT mice never revealed aberrant tubules.

A distinguishing feature of the double KO mice was that the aberrant tubules and the main duct were closely apposed to each other with no intertubular space existing between the two (Fig 4C–4F). In fact, at times the aberrant tubules impinged upon tubules of the main epididymal duct such as to form deep indentations in them (Fig 4C). At this site the epithelial cells of the main duct were often disfigured and reduced in size, while the remainder of the main duct retained its epithelial structural features. Spermatozoa were abundant in the lumen of the main epididymal duct (Fig 4C). There was no indication that the aberrant tubules were in continuity with the main duct, as spermatozoa were absent consistently from their lumen (Fig 4C and 4F). Myoid cells completely enclosed aberrant tubules and were shared by the main epididymal duct at areas where the two where closely apposed to each other (Fig 4C–4E). A highly convoluted and multilayered basement membrane surrounded the epithelium of the aberrant tubules and the main duct (Fig 4B and 4F).

#### Corpus epididymidis

**Fig 5:**
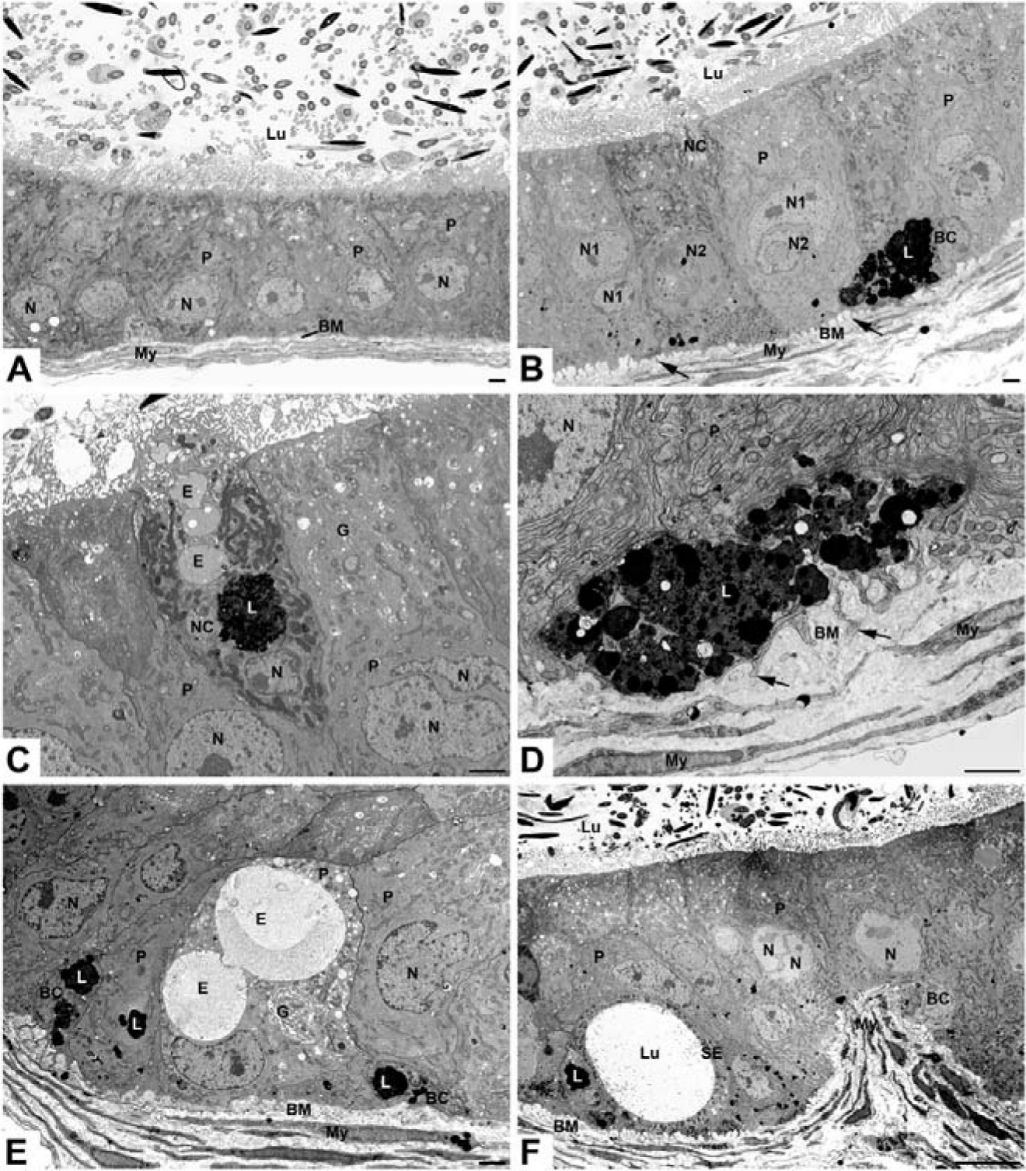
EM of the corpus epididymidis of WT (**A**) and *neu3*^*-/-*^*neu4*^*-/-*^ (**B-F**) mice. In (**A**), P cells reach the Lu and contain Spz. Nuclei (N) are more or less spherical and regular in appearance. A thin BM underlies the epithelium and several My layers are seen. In (**B**), P cells reach the Lu where Spz are plentiful. Nuclei (N) of P cells are highly irregular in form. One N encircles organelles of the cytoplasm (N2). Some P cells contain more than one nucleus (N1). A BC is filled with huge L. The base of P cells and a BC shows small thin foot-like processes (arrows) that project into a highly convoluted BM. In (**C**) a NC is filled with large endosomes (E) and L. In (**D**) a BC filled with huge L reveals thin foot-like processes (arrows) that project into a highly anastomotic BM. In (**E**), gigantic endosomes (E) and L appear in the cytoplasm of a P cell revealing an elaborate G. In (**F**), a thin continuous squamous circular epithelial cell (SE) is noted surrounding a large Lu revealing microvilli but absence of Spz. The SE contacts the BM by thin foot-like processes. The Lu of the main duct contains Spz. In (**E**) and (**F**) a large L appears in the infranuclear cytoplasm of a P cell, one of which is binucleated. Scale bars: Scale bars: **A-E** = 2μm; **F** = 10μm.

In the corpus epididymidis of WT mice, columnar principal cells reached the lumen and contained spermatozoa and their nuclei were more or less spherical and regular in appearance. A thin basement membrane underlies the epithelium and several myoid cell layers were evident (Fig 5A). In *neu3*^*-/-*^*neu4*^*-/-*^ mice, columnar principal cells reached the lumen where spermatozoa were plentiful, but nuclei were at times highly irregular in form and encircled organelles of the cytoplasm (Fig 5B). Some principal cells appeared to contain more than one nucleus. Basally located cells were evident and filled with huge dense lysosomal structures (Fig 5B and 5D), as were narrow cells (Fig 5C). On occasion, principal cells contained gigantic vacuoles that filled their cytoplasm (Fig 5E). The base of principal and basally located cells showed many small thin foot-like processes that projected into a highly convoluted, anastomotic multilayered basement membrane (Fig 5B, 5D and 5E). On occasion what appeared to be a highly vacuolated epithelial cell embedded itself within the base of the epithelium of the normal duct (Fig 5F). The vacuolated cell appeared as a thin continuous squamous circular layer surrounding a large lumen (Lu) revealing microvilli but absence of spermatozoa. The vacuolated cell contacted the basement membrane by thin foot-like processes (Fig 5F).

#### Cauda epididymidis

**Fig 6:**
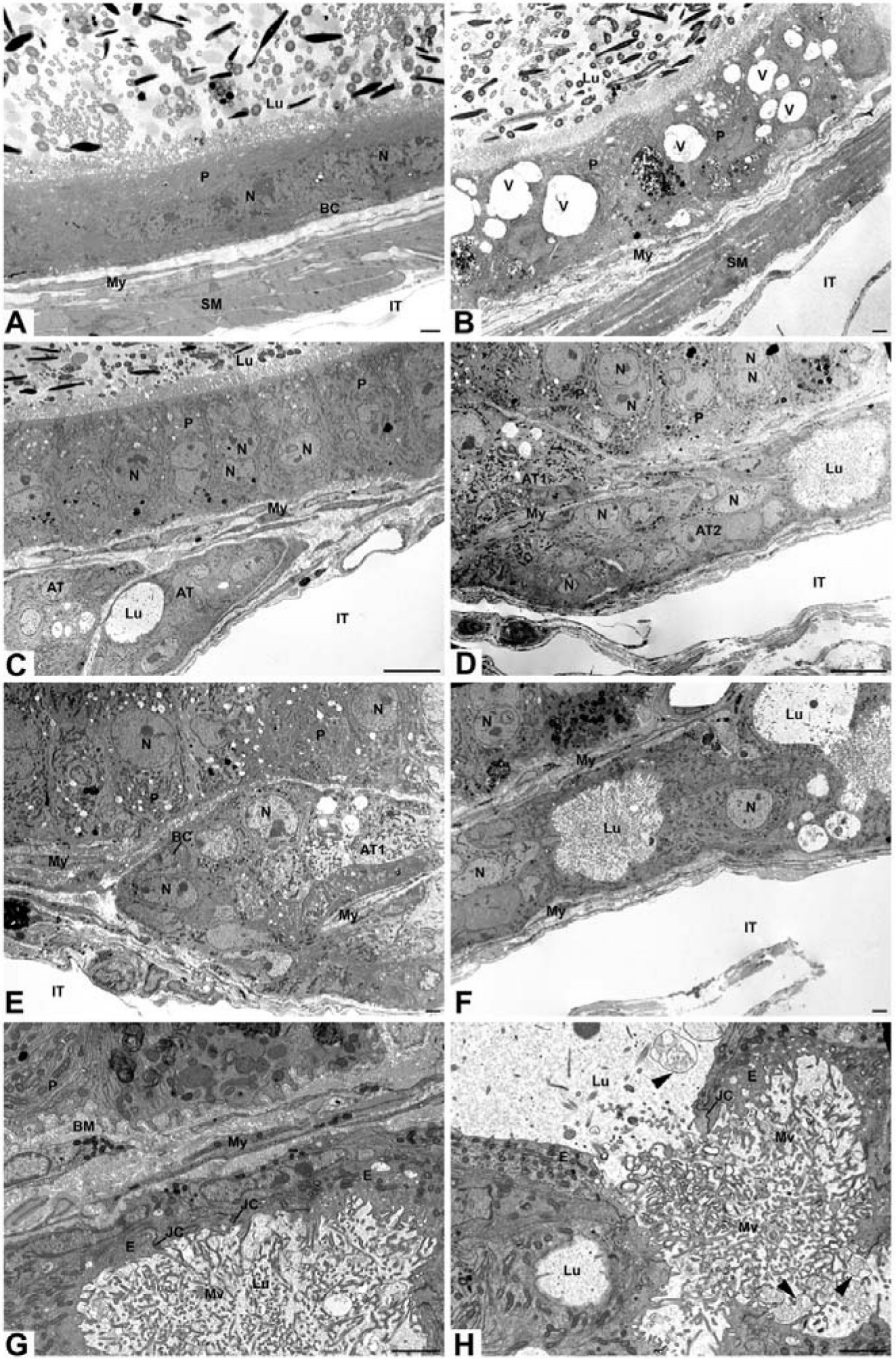
EM of the distal cauda epididymidis of WT (**A**) and *neu3*^*-/-*^*neu4*^*-/-*^ (**B**) mice. In (**A**), P cells show large irregularly shaped N and the epithelial cells envelop a Lu containing Spz. In (**B**), P cells contain several huge pale stained vacuoles (V) and large L filled with a dense granular material. Images **(C-H**) show the proximal cauda of *neu3*^*-/-*^*neu4*^*-/-*^ mice. In (**C**), adjacent ATs are situated parallel to normal tubules. The latter shows differentiated P cells, some with a double nucleus (N). Both ATs consist of a mass of undifferentiated cells, but the AT on the right reveals a Lu. Its encasing cells are highly attenuated with microvilli projecting into the Lu, which is devoid of Spz. The ATs are enveloped by My cells, which are shared with the normal tubule. Image (**D**) is a low power magnification of an AT in proximity to a normal duct. Part of an AT (AT1) impinges on a normal tubule, while the remainder of the AT (AT2) stretches beneath and parallel to the normal tubules. AT1 consists of a congealed mass of cells, while cells of AT2 at the far right form a distinct Lu containing microvilli. Details of AT1 and AT2 are shown in (**E**) and (**F**). In (**E**), one of the ATs (AT1) reveals a mass of cells that appear to show some differentiated organelles that in part resemble P cells of the normal tubule. No Lu is evident in AT1, but a My cell projects itself into the mass of cells seemingly to separate in part AT1 from AT2. In (**F**) the AT labeled AT2 in image (**D**) is further investigated. On the left side of the AT2 tubule, cells of different shapes and sizes, some of which appear to be squamous, border a Lu. On the right side of the field, a confluence of the Lu of 2 ATs appears to be taking place. Images (**G**) and (**H**) are a high power magnification of (**F**) and show flat undifferentiated epithelial cells (E) bordering a Lu and revealing JC. The Lu contains microvilli (Mv) and membranous whorls (arrowheads) but no Spz. Scale bars: **A, B, E, F, G** and **H** = 2μm; **C** and **D** = 10μm.

In WT mice, the cuboidal principal cells revealed large irregularly shaped nuclei and several small lysosomes. The epithelial cells enveloped a large lumen with spermatozoa (Fig 6A). In *neu3*^*-/-*^*neu4*^*-/-*^ mice, some principal cells contained several prominent huge pale stained apical and supranuclear vacuoles containing membranous profiles, in addition to large lysosomes filled with a dense granular material (Fig 6B). Some nuclei of principal cells were binucleated. Spermatozoa were plentiful in the lumen (Fig 6B). In the case of the cauda, aberrant tubules were evident but they were mainly seen extending alongside the main epididymal duct (Fig 6C–6F). These aberrant tubules presented two phenotypes. Some appeared to be isolated congealed masses of cells without evidence of a lumen; the cells forming these masses had a degree of differentiation as noted by the presence of organelles that were fairly prominent such as endosomal elements, a Golgi apparatus and ER cisternae; basal cells were evident in these masses (AT1, Fig 6E). On the other hand, other congealed masses of cells along their length revealed a distinct lumen (AT2, Fig 6D and 6F). In fact, there was a distinct continuity of cells of the congealed mass and the site where a lumen was evident. At the site of the lumen the bordering cells were attenuated and without a differentiated appearance (Fig 6F–6H). Junctional contact points between adjacent cells at their apices were evident (Fig 6G and 6H). Images of the apparent fusion of the lumen of 2 adjacent aberrant tubules were noted (Fig 6H). No spermatozoa were present in the lumen of these aberrant tubules, but membranous bodies and microvilli were evident (Fig 6C, 6D, 6F, 6G and 6H). The mass of congealed cells including those that presented a lumen were enveloped by myoid cells, which were shared with those of the main epididymal duct in areas where the two approximated one another (Fig 6D, 6E, 6F and 6G). Myoid cells at times separated in part the congealed masses (Fig 6E).

#### Prosaposin immunostaining

**Fig 7:**
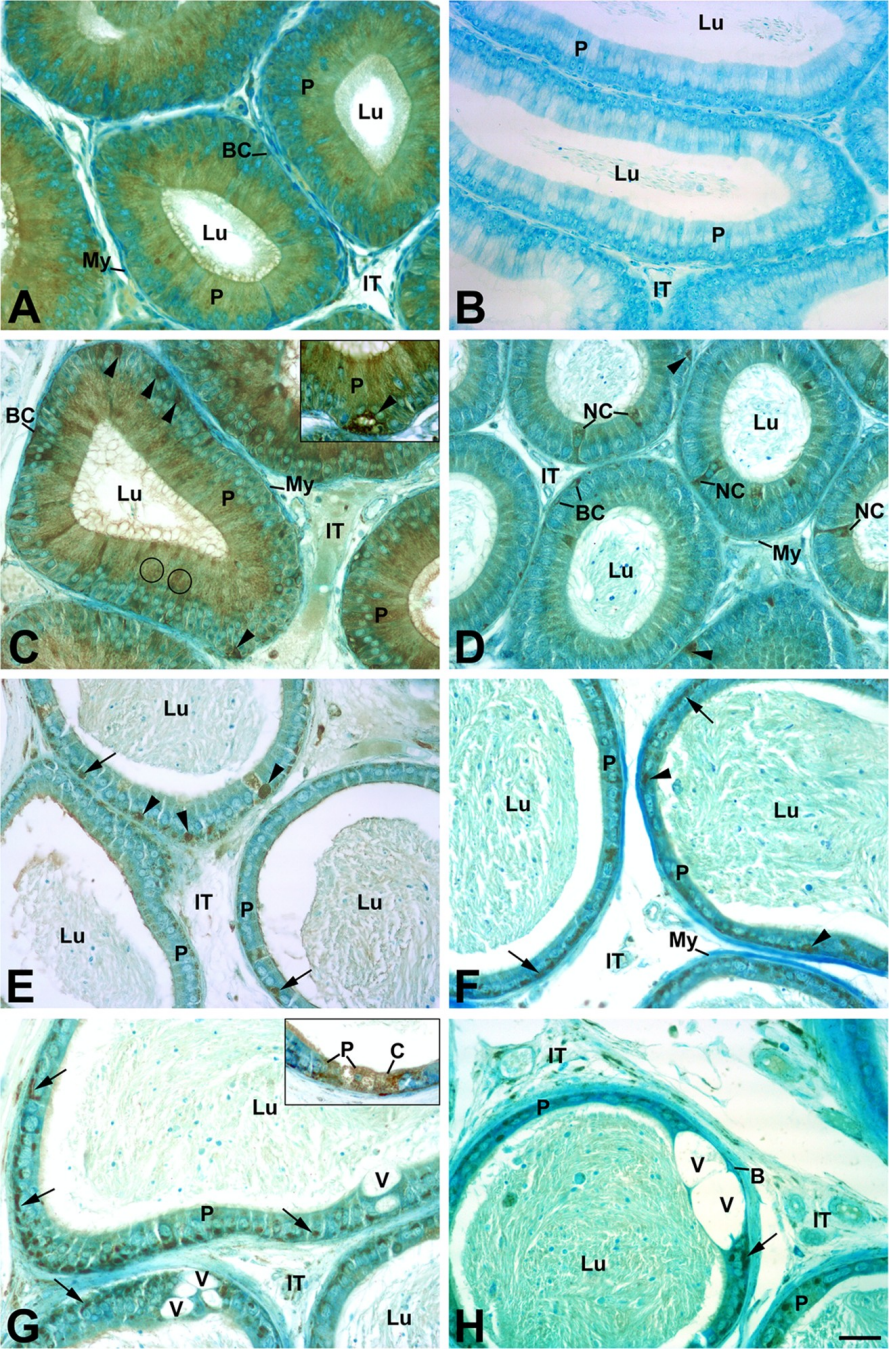
Immunolocalization of Prosaposin in the epididymis of *neu3*^*-/-*^*neu4*^*-/-*^ mice. Initial segment in (A) shows immunoreaction of prosaposin in wild type mice. (B) shows negative control. Immunoreaction of Prosaposin in the initial segment (**C**), caput (**D**), proximal (**E**) and distal (**F-H**) cauda regions of the epididymis of *neu3*^*-/-*^*neu4*^*-/-*^ mice immunostained with an anti-prosaposin antibody. In (**C**), small punctate reactive lysosomes (circles) are noted in the supranuclear area of P cells and in thin elongated processes of a BC. Large highly reactive cells (arrowheads) are seen at the base of the epithelium, as also demonstrated in (**C inset**). In (**D**), a narrow cell (NC) is reactive as well as several small (arrows) and large basally located cells (arrowheads). In (**E-H**) several large highly reactive cells (arrowheads) are noted at the base of the epithelium. In (**E-G**), P cells reveal infranuclear reactive L at their base (arrows). In (**G and H**) large vacuoles (V) are seen in the epithelium as well as basally located cells. In (**G inset**), P and clear (C) cells are reactive for prosaposin. Scale bar: 20μm.

In all regions of the epididymis of *neu3*^*-/-*^*neu4*^*-/-*^ mice, an intense staining was noted with anti-prosaposin antibody. This was the case for basally located cells of all epididymal regions (Fig 7A–7F), supranuclear lysosomal structures in principal cells (Fig 7A), narrow cells (Fig 7B), and infranuclear lysosomes of principal cells of the cauda region (Fig 7C–7E). Large vacuolations (V) were often noted in the cauda region (Fig 7E and 7F) that could imply degeneration of epithelial cells. Prosaposin staining has been demonstrated in adult rats where reactions were noted in lysosomes of epithelial epididymal cells [62].

## Discussion

In the present study several major findings were noted in the double *Neu3* and *Neu4* KO mouse model. One was the observation of a lysosomal accumulation in epithelial cells of the efferent ducts and epididymis. Neuraminidases (Neu) are a family of 4 enzymes involved in the removal of sialic acid residues from glycoconjugates [48, 49], with both Neu3 and Neu4 localizing to endosomes and lysosomes where they are involved in in lysosomal catabolism [51, 53–56]. In the absence of either one of these enzymes, the specific ganglioside substrate accumulates in lysosomes, leading to the development of a lysosomal storage disorder [50, 51, 57, 58] as noted in the nervous system which in turn causes neuroinflammation, lipofuscin accumulation and astrogliosis leading to learning impairment and memory loss [59]. The development of these conditions thus highlights the importance of Neu3 and Neu4 in the catabolism of gangliosides.

### Lysosomal storage disease in epithelial cells of the efferent ducts and epididymis

In the efferent ducts, the nonciliated cells of *neu3*^*-/-*^*neu4*^*-/-*^ mice revealed the presence of large dense spherical structures as well as large empty looking apical and supranuclear vacuoles. Is well documented that the nonciliated cells are highly active in the uptake of proteins from the lumen and contain numerous spherical lysosomes [4, 24, 63]. In different lysosomal KO models such as cathepsin A, Hexosaminidase A, and prosaposin lysosomes accumulate in the cytoplasm and take on gigantic sizes, some of which take on a pale appearance and which have been confirmed to be lysosomal in nature by EM immunocytochemical analyses [39–42, 47, 64]. Notable, however, was the finding of long irregularly shaped dense cylindrically shaped structures that at times stretched across the cytoplasm from the apical to the supranuclear area of the cell. What was peculiar was the finding of linear rows, at times parallel to each other, of small to medium sized empty looking spaces in the matrix of these structures. Small dense lysosomes appeared to be fused with these structures and were stuck to their outer wall. Cylindrical structures with this type of phenotype have not been reported in epithelial cells of the efferent duct or epididymis of other lysosomal storage diseases [39–42, 47, 64]. While it is possible that the spaces correspond to cristae within the mitochondrial matrix, the absence of membranes surrounding them is conspicuous. The fusion of lysosomes with the cylindrical structures may account for the internal degradation of the membrane of cristae leaving behind the empty looking spaces. Mitochondria are known to be large branching organelles and in *neu3*^*-/-*^*neu4*^*-/-*^ mice their shape and internal features may be altered. What is relevant is that Neu4 while localized to lysosomes has also been shown to be present in the mitochondrial lumen [51, 54–56]. It is equally possible that these cylindrical structures represent lysosomes suggesting a unique internal appearance of their contents caused by the absence of degradation by Neu3 and Neu4. For the time being, the identification of these cylindrical structures awaits further investigation.

In the epididymis, lysosomal abnormalities were region-specific. In the initial segment, small to medium sized lysosomes were evident in the supranuclear area of principal cells as revealed by prosaposin staining. However, in the cauda region, infranuclear lysosomes accumulated in principal cells as revealed by intense prosaposin reactions. Narrow and basally located cells were also highly reactive for prosaposin. Such findings are reminiscent of those noted in other lysosomal KO mouse models [39–42, 47, 64]. The epithelium especially in the cauda region exhibited large to gigantic pale vacuoles that suggested a loss of epithelial cells.

Throughout the epididymis, basally located cells were present, but many were of large size compared to WT mice and highly reactive for prosaposin. They were highly reactive for prosaposin. In the EM, they exhibited large dense lysosomal elements, some of which had a distinct lipidic nature suggesting they correspond to lipofuscin granules. The field of basally located cells has become intriguing with the identification not only of keratin 5 marked classic basal cells, but also epididymal mononuclear phagocytes (F4/80 macrophages as resident epithelial cells and CD11c+ (integrin alpha X chain) dendritic cells), all sharing contacts with the basement membrane. In our *neu3*^*-/-*^*neu4*^*-/-*^ mouse model severe alterations were noted in basally located cells suggesting uptake of substrates that could not be degraded owing to the absence of Neu3 and Neu4. Hence these cells follow the conventional theme of a lysosomal storage disease for the *neu3*^*-/-*^*neu4*^*-/-*^ mouse model. Currently it is reasonable to suggest that the affected basal cells correspond to epididymal mononuclear phagocytes, but confirmation of this would require more detailed analyses using appropriate markers for these cells. The discovery of a functional population of epididymal mononuclear phagocytes in the epididymal epithelium has raised questions regarding their role in clearing defective epithelial cells in the steady-state epididymis, as well as pathogens and abnormal spermatozoa in the lumen [15–17].

### Effects of intercellular spaces in the epithelium of the initial segment

In the initial segment, large dilated intercellular spaces were evident especially near the base of the epithelium in *neu3*^*-/-*^*neu4*^*-/-*^ mice. These spaces were filled with variable sized vesicular elements as well as membranous profiles. In addition, numerous capillaries were prominent some very large. The initial segment is exemplified by having the tallest epithelial cells, a small lumen, highest blood flow of any region [65], and being surrounded by a dense subepithelial network of fenestrated capillaries [66–68].

The initial segment is known to move fluids from the lumen to the interstitium owing to numerous aquaporin proteins residing on the microvilli of principal cells of this region [69, 70]. This function serves to concentrate spermatozoa in the epididymal lumen and allow more effective interactions of secreted proteins with the surface of spermatozoa [2]. The integrity of these spaces may be altered due to increased movement of fluid from the lumen to the underlying interstitium and capillary networks, which appear as a consequence of absence of Neu3 and Neu4. Distended intercellular spaces and vacuoles have been noted when the rat cauda epididymidal lumen is perfused with high Na and low Na, respectively [71]. Micropuncture studies could address such parameters in the absence of Neu3 and Neu4 in future studies.

### Effects on basement membrane and interstitium

While the epididymis consists of a tube enclosed of epithelial cells enveloping a central lumen, the underlying interstitium is formed of the basement membrane, a homogeneous layer of filamentous nondescript material that surrounds and is in close contact with the epithelium, layers of myoid cells and the intervening space between adjacent epididymal tubules containing blood and lymphatic channels, blood cells and collagen, fibrocytes and occasional blood derived cells. Recently, the interstitium, a component of many organs of the human body, has been defined as a novel organ. It had completely eluded scientists as a distinct organ but has been demonstrated to have functions related to normal and disease states [72]. Another striking feature of the *neu3*^*-/-*^*neu4*^*-/-*^ mouse model was the dramatic appearance of the basement membrane. It was highly convoluted and thicker than that of WT mice. Vesicular profiles were noted in the anastomotic spaces of the highly tortuous basement membrane. The increased size of the basement membrane has been demonstrated in the epididymis of old animals and after radiation [6, 73, 74] and suggests that the epithelium is in need of a more consolidated bond with the underlying interstitium. Notably, many thin foot-like processes of the epithelial cells (principal and basally located) were often noted that appeared to anchor these cells to the basement membrane. Myoid cells reside beneath the basement membrane and act as a means to move the immotile spermatozoa down the epididymal duct [75]. They form concentric layers, which gradually increase from the initial segment to the cauda region. While they contain lysosomes they did not appear to be affected in *neu3*^*-/-*^*neu4*^*-/-*^ mice. In all epididymal regions, it was not uncommon to find numerous immune cells with characteristics of macrophages, lymphocytes, and monocytes in the intertubular spaces. It is presumed that these cells migrate from the circulation to this site owing to the epithelial cell abnormalities caused by the double KO mouse model and which has been demonstrated in cathepsin A KO mice [64].

### Formation of aberrant tubules

The epididymal duct precursor, known as the Wolffian duct, arises within the urogenital ridge during embryogenesis. Transformation of the straight Wolffian duct into the complex and coiled components of the male reproductive tract requires synchronized elongation and coiling and likely involves a combination of cell proliferation, cell shape changes, cell rearrangements, fluid secretion, apoptosis and/or possible cell division “hotspots” that contribute to coiling [76, 77]. Moreover, the epididymis is noted for the fact that there is a lack of branching morphogenesis of the main duct during development.

What was surprising in the double *Neu3/Neu4* KO mouse was the presence of aberrant tubules deeply indenting tubules of the main duct with which they shared myoid cells. Others lay parallel to the main duct stretching between the myoid cell layers. No such tubules were ever noted in WT mice. Aberrant tubules were observed both as a dense mass of congealed cells containing basal cells but no lumen, as well as small tubules with a lumen revealing apical junctional contact points and basal cells. However, while cross sectional tubular epididymal profiles of the main duct were always well separated by a large interstitial space in WT mice, in all cases, aberrant tubules were not separated from tubules of the main epididymal duct by such a space. It is presumed that cell proliferation is the major contributor of Wolffian duct growth and impacts not only in duct lengthening but also duct thickening [78] both of which are highly coupled to coiling ability. Fluid secretion into the lumen has also been considered to be a driver of tubule growth [79–81] and since a patent lumen is observed in the mouse Wolffian duct around day E13-14 [78], it would indicate that fluid is secreted into the duct at this time. Indeed day E13-14 is a critical time point with the main duct beginning to elongate and coil.

The presence of aberrant tubules, some with a distinct lumen, residing adjacent to those of the main duct suggests that during E13-14 and in the absence of Neu3 and Neu4, the main epididymal duct is unable to maintain the uniform integrity of its elongation and coiled properties. Sialic acids are acidic monosaccharides typically found at the outermost ends of the sugar chains of animal glycoconjugates. By virtue of their negative charge, they potentially can inhibit intermolecular and intercellular interactions [82]. In the double KO mouse, failure to remove sialic acids during epididymal duct development may result in lack of recognition and adhesion of some of its epithelial cells undergoing proliferation and coiling resulting in aberrant tubule formation. Sialic acids are terminal acidic monosaccharides found on glycoproteins and glycolipids. Sialic acids function as crucial recognition markers in multicellular organisms where they mediate a variety of biological phenomena. Indeed Neu3 modulates plasma-surface biological events and plays a pivotal role in controlling transmembrane signaling for different cellular processes, including cell adhesion, recognition and differentiation [48–53].

While aberrant tubules are formed in *neu3*^*-/-*^*neu4*^*-/-*^, several findings suggest that they do not remain connected to tubules of the main duct. One is the fact that the cells lining aberrant tubules with a lumen are undifferentiated. Their undifferentiated appearance would result from absence of lumicrine factors (testicularly derived luminal fluids, proteins, spermatozoa, and luminally-derived androgens) entering their lumen. These factors have been shown to be critical for differentiation of epithelial cells [83–88]. Secondly, no spermatozoa were ever noted in the lumen of aberrant tubules, which is not the case for the main epididymal duct. Studies employing mice at different time points during development revealed that prior to postnatal day 15, the initial segment was lumicrine factor-independent [89] and this therefore would also include spermatozoa. Hence aberrant tubules appear to have formed prior to postnatal day 15. Future experiments of mice at earlier postnatal days and possibly prenatal days would be in order to more precisely determine when the aberrant tubules begin to form.

What is also intriguing is the presence of small basal cells in the aberrant tubules with or without a lumen. While their identity is unknown, as is their source of origin, it may be suggested these cells arising early during development would have important functions to fulfil in the main epididymal duct had these tubules not faltered. In fact, the finding that aberrant tubules are formed due to absence of Neu3 and Neu4, and that such tubules do not become fully differentiated could be of interest to scientists involved in formation of organoids from isolated epididymal cells. Moreover, the aberrant tubules reveal basal cells and these have been implicated as stem cells of the epididymis [90]. The fact that the aberrant tubules contain basal cells points to their importance in tubular formation, and this double Neu3 and Neu4 model may serve to be useful for identifying cells and related factors involved in early tubular formation.

Another point to consider is whether or not the aberrant tubules are related to congenital anomalies noted in the human epididymis. These comprise the appendix testis (sessile hydatid of Morgagni), the appendix epididymis (pedunculated hydatid of Morgani), the aberrant ducts (Haller's organs) and paradidymis (Giraldé's organ or Henle's para-epididymis) [91]. It would be interesting to determine if such anomalies are related to absence of neuraminidase activity.

In the epididymis of *neu3*^*-/-*^*neu4*^*-/-*^ mice, the epithelium occasionally revealed the presence of thin continuous circular squamous epithelial cells. These cells appeared to surround a large lumen (Lu) containing the projecting microvilli of the squamous cell, but complete absence of spermatozoa. Such cells have been noted in ligated animals of the mouse [92], but their origin or functional significance are unknown.

While the *neu3*^*-/-*^*neu4*^*-/-*^ mice reveal lysosomal abnormalities in epithelial cells and aberrant tubules, it is evident that they still retain a functional epididymis with tubules of the main duct being structural intact as they contain ample spermatozoa and are fertile (Pshezhetsky’s lab, unpublished data). Taken together, the finding of aberrant tubules as small offshoots formed of undifferentiated cells and lacking spermatozoa alongside the main epididymal duct suggests a guidance miscue of epithelial cells engaged in formation of the main duct during early embryonic development. Thus, for the first time the importance of Neu3 and Neu4 has been uncovered as a means to ensure the proper normal development of the highly coiled epididymal duct.
